# Partially substituting roughage with traditional Chinese herbal medicine residues in the diet of goats improved feed quality, growth performance, hematology, and rumen microbial profiles

**DOI:** 10.1186/s12917-024-04412-1

**Published:** 2024-12-23

**Authors:** Yong Long, Naifeng Zhang, Yanliang Bi, Tao Ma, Pramote Paengkoum, Jiamin Xin, Wen Xiao, Yanpin Zhao, Chao Yuan, Defeng Wang, Yang Yang, Chaozhi Su, Yong Han

**Affiliations:** 1 Guizhou University of Traditional Chinese Medicine, Guiyang, 550025 China; 2 Institute of Feed Research of Chinese Academy of Agricultural Science, Beijing, 100081 China; 3 School of Animal Technology and Innovation, Institute of Agricultural Technology, Suranaree University of Technology, Nakhon Ratchasima, 30000 Thailand; 4 Institute of Animal Husbandry and Veterinary Sciences, Guizhou Academy of Agricultural Sciences, Guiyang, 550025 China; 5 The First Affiliated Hospital of Guizhou, University of Traditional Chinese Medicine, Guiyang, 550001 China

**Keywords:** Apparent digestibility, Feed quality, Growth performance, Traditional Chinese herbal medicine residues, Rumen microbes, Serum biochemical indicators

## Abstract

This study aimed to reveal the effect of traditional Chinese herbal medicine residues (TCHMR) on growth performance, hematology, ruminal microbiota, and economic benefits of Guizhou black male goats through the fermented total mixed ration (FTMR) diet technique. A total of 22 Guizhou black male goats with an initial weight of 21.77 ± 0.85 kg were randomly divided into 2 groups (*n* = 11), with 11 goats in each group. The control group (CON) was fed a traditional total mixed ration (TMR) diet without the TCHMR. The TCHMR group was fed an FTMR diet containing 40%TCHMR. Compared with the CON group, the results showed that the incorporation of TCHMR into goat diets reduced feeding costs and Feed conversion ratio (FCR). On the contrary, it improved (*P* < 0.01) feed quality, apparent digestibility of Dry matter (DM), Crude Protein (CP), Neutral detergent fiber (NDF), average daily gain (ADG), and dry matter intake. Interestingly, TCHMR also reduced (*P* < 0.01) acetate levels in the rumen of goats. Supplementally, TCHMR significantly increased (*P* < 0.01) the levels of GH, IgM, IgA (*p* < 0.05), and IFN-γ (*P* < 0.05), while significantly reducing (*P* < 0.01) the levels of IL-6, ALT, and AST in serum. Notably, at the phylum level, TCHMR significantly reduced (*P* < 0.01) the abundance of *Bacteroidota* and increased (*P* < 0.01) the abundance of *Firmicutes*. Moreover. at the genus level, TCHMR significantly reduced (*P* < 0.01) the abundance of *Prevotella*, *F082*, and *Bacteroidales_RF16_group*, while *Muribaculaceae*, *Proteus*, *Lachnospiraceae_ND3007_group*, and *Ruminococcus* were increased (*P* < 0.01). In conclusion, our current findings indicated that 40% TCHMR improved feed quality and the apparent digestibility of nutrients. Additionally, 40% TCHMR improved the growth performance and immunity of Guizhou black male goats, while also reorganizing the composition of ruminal microbiota. So far, under the conditions of this experiment, we have not found any negative effects of 40% TCHMR on goats. This study will be a new idea for developing feed resources, which will reduce environmental pollution and the cost of animal husbandry.

## Introduction

China has more than a thousand years of traditional Chinese herbal medicine culture and is rich in Chinese medicinal materials resources. There are more than 1,500 traditional Chinese herbal medicine companies in China [63]. The area covered by the artificial cultivation of Chinese medicinal materials has exceeded 2.4 million hectares, and the annual output of Chinese medicinal materials is about 70 million tons. When effective functional components are extracted from Chinese medicinal materials, a large amount of traditional Chinese herbal medicine residues (TCHMR) will be produced, with the annual output of residues exceeding 35 million tons [75]. The main treatment methods for TCHMR are incineration, stacking, and burial. Incineration releases medicinal gases that pollute the air, while stacking, especially during the rainy season, easily leads to decay, contaminating soil and rivers. Moreover, decaying stacked TCHMR emits a mixture of medicinal gases, which not only pollute the air but may also pose risks to human health. Burial, notably, causes more significant soil pollution, as the complex drug compounds can hinder plant growth in heavily contaminated areas, leading to environmental degradation and resource wastage , [23, 43, 47]. At present, the treatment methods of TCHMR are mainly in the extraction of active resources [25], cultivation of edible mushrooms [29], production of animal feed [62], production of renewable energy [73] and wastewater treatment [60].

TCHMR contains many sugars, flavonoids, alkaloids, terpenes, and other biologically active substances. It also contains nutrients such as crude protein (CP), crude fiber (CF), ether extract (EE), and mineral substances, and has certain medicinal effects and feeding value [38]. TCHMR can be directly added to the feed or added to the feed after microbial fermentation to feed animals [1]. A previous study by Liang et al. [39] indicated that the incorporation of TCHMR in goat diets could increase the level of immune globule and had a positive effect on the rumen fermentation and energy metabolism of sheep. However, due to the high content of TCHMR anti-nutritional factors, it can directly affect the absorption and utilization of nutrients by animals, and some TCHMR have a bad taste. Unreasonable additions or improper treatment methods will directly affect the palatability of diets and reduce animal feed intake [38].

Through investigation, we found that the feed utilization research related to TCHMR is still in the basic experimental stage, and there were no related feed products. Developing a cost-effective and medicinal feed to replace the use of antibiotics and lower feeding costs has long been one of our primary research goals. Previously, our research team conducted a field single-cage feeding test, and different proportions (30%, 40%, 50%, and 60%) of TCHMR (Including *Panax ginseng*, *Codonopsis pilosula, Plantago asiatica*, *Astragalus membranaceus*, *Eleutherococcus senticosus*, *Mylabris*, and so on.) The chemical composition of TCHMR is moisture (55.89 ± 1.26%); dry matter (DM) (92.45 ± 0.82%, air-dry basis), CP (10.35 ± 0.22%), crude ash (Ash) (3.26 ± 0.01%), neutral detergent fiber (NDF) (70.31 ± 0.25%), EE (1.04 ± 0.18%), calcium (Ca) (1.83 ± 0.01%), and phosphorus (P) (0.13 ± 0.00%)) were added to the total mixed ration (TMR) of goats for fermentation treatment, and fermented total mixed ration (FTMR) was prepared for feeding Guizhou black male goats. We found that the addition of 40% TCHMRFTMR group had the best effect in improving the feed quality and feeding behavior, promoting rumination activities, and reducing the diarrhea rate of Guizhou black goat-growing male lambs [45]. From this, we believe that long-term supplementing of 40% TCHMR FTMR may have many positive effects on growth performance, hematology, ruminal microbiota, and economic benefits Guizhou black male goats. Therefore, the goal of the current study was to conduct a more comprehensive reveal of the effects of 40% TCHMR on the growth performance, rumen fermentation, hematology, and ruminal microbiota of Guizhou black male goats. Ultimately, TCHMR has the potential to be developed into a cost-effective medicinal feed that can both replace antibiotics and lower feeding costs.

## Materials and methods

### Animals, Materials, experimental design, diet, and management

All animals in this study were provided by the Institute of Animal Husbandry and Veterinary Sciences, Guizhou Academy of Agricultural Sciences (Guiyang, China). TCHMR was provided by Guizhou Yibai Pharmaceutical Co., Ltd. (Guiyang, China) and obtained on the second day after delivery. White‑rot fungi were provided by the Beijing Microbiological Culture Collection Center (Beijing, China). The mixed fermentation agent (lactic acid bacteria, *Bacillus licheniformis*, yeast, *Bacillus subtilis*, and so on, the number of live bacteria is ≥ 5 × 10^11^ CFU/g) were purchased from Luoyang Oukebaik Biotechnology Co., Ltd. (Luoyang, China). The mixed enzyme preparation (Contains cellulase ≥ 7 000 U/g, xylanase ≥ 30 000 U/g, β-glucanase ≥ 12 000 U/g, and pectinase ≥ 3 000 U/g) was purchased from CJ Youtel Biotechnology Co., Ltd. (Shanghai, China).

The TMR formulation was redesigned based on the optimum TCHMR level from a previous study [45]. A total of twenty-two healthy, 6-month-old Guizhou black male goats, with an initial body weight of 21.77 ± 0.85 kg were selected and divided into 2 groups (*n* = 11) by randomized complete block design (RCBD). Each group consisted of 11 goats. The control group (CON) was fed a TMR diet without the TCHMR. The TCHMR group was made into FTMR by adding 40% TCHMR according to the designed formula and adding mixed starter, white‑rot fungi, and mixed enzyme preparation according to the product usage method recommended by the company, using fermentation bags to compact, and ferment at the room temperature and seal for 15 days. The experimental period spanned 75 days, comprising 15 days of adaptation followed by 60 days dedicated to data and sample collection. All diet compositions and nutrient levels were based on the nutritional requirements of NRC (2007) [53] (Table 1).

**Table 1:** Approximate composition and nutritional levels of diet (%, air-dry basis)

Ingredient	Group	
	CON	TCHMR
Corn	28.00	26.00
Wheat bran	7.00	-
Rice bran	9.10	4.22
Soybean meal 43	4.50	7.50
Rapeseed meal	3.00	4.40
Edible oil	-	1.3
NaCl	0.50	0.50
1% composite premix ^a^	0.46	0.46
Urea	1.64	0.62
Cane molasses	-	2.00
Peanut vine	45.80	13.00
Traditional Chinese herbal medicine residues	-	40.00
Total	100.00	100.00
Chemical composition^b^		
DM	80.73	57.51
CP	20.82	20.72
NDF	39.56	28.58
Ca	0.58	1.03
P	0.50	0.44
ME, MJ/kg	11.61	11.61

*P* Phosphorus, *ME* Metabolizable energy

^a^ Premix provides per kg of ration: Vitamin D, 15,000 IU; Vitamin A, 10,000 IU; Vitamin E, 200 IU; Zinc, 5000–3750 mg; Iron, 1000–7500 mg; Copper, 140–150 mg; Manganese, 600–3000 mg; Iodine, 5–200 mg; Selenium, 3.5–10 mg; Cobalt, 4–40 mg; Ca, 4–20% and P, 2–8%

^b^ Except for the energy value, the remaining chemical components are actual measured values

Each goat was used individually in a stainless steel, fully automated precision feeding system consisting of automatic weighing, temperature detection, humidity detection, automatic water supply, safety sensing, and hydrogen sulfide systems. This fully automatic precision feeding system comprised 44 metabolic cages (2m^2^). Before the experiment, it was comprehensively sterilized, dewormed, and immunized in accordance with the farm's standard procedures. The feeding time was 9:00 and 17:00 each day. Adjust the daily feed amount based on the remaining feed from the previous day and ensure that the remaining feed amount before each feeding is 10% of the last feeding.

### Organic acid, Ammonia nitrogen (NH_3_-N), pH, and microbial population analysis in FTMR

Each experimental group had 6 replicates. The feed was put into a vacuum bag (30*25cm) and the excess air was extracted by a vacuum machine (VM400E/B, Wenzhou Brother Machinery CO. LTD, Zhejiang, China) so that the sample was fermented under vacuum. After 15 days of feed fermentation, 25g samples were accurately weighed for each repeat, and mixed with 225mL of sterile water, incubated the mixture overnight in a 4°C refrigerator, and finally filtered through 4 layers of gauze. The filtrate was used to detect pH (PHS-3E, Shanghai Leici Instrument Co., Ltd., Shanghai, China), NH_3_-N, lactic acid, and volatile fatty acid (VFA) (Including acetate, propionate, butyrate, and so on.) [52]. NH_3_-N content detection was based on the colorimetric procedures of hypochlorite and phenol [8]. The method for analyzing water-soluble carbohydrates (WSC) in feed followed the method outlined by Johnson et al. [30]. HPLC and GC–MS were used to analyze the concentration of lactate and the content of VFA, respectively. The plate culture approach was used to analyze the microbial population. Lactic acid bacteria were cultured on Man, Rogosa, and Sharpe (MRS) agar medium, whereas molds and yeasts were cultured using Rose Bengal agar mediums [17]. All culture media in this experiment came from Guangdong Zhongshan Bai Microbiology Technology Co., Ltd. Zhongshan, China. The viable count of microorganisms in colonies was counted in colony-forming units (cfu)/g of fresh matter (FM). For changes in chemical composition after TCHMR TMR fermentation, please refer to previous research reports [46].

### Growth performance and economic benefits

Before each feeding during the test, the leftover feed was cleaned and weighed. Furthermore, the average daily gain (ADG) is calculated by transmitting the weight of the goats to the computer regularly every day through the precision feeding system during the test. The method of calculating dry matter intake (DMI), feed conversion ratio (FCR), feed weight gain cost and weight gain benefit in feed follows the approach outlined by Long et al. [46].

### Apparent digestibility

The digestibility trial lasted for 12 days, comprising a 7 days adaptation period and a 5 days period for whole fecal collection. About 10% of the total feces from each goat was extracted per day and stored until it was completely mixed after collection. Subsequently, after adding 10% diluted sulfuric acid to stabilize the nitrogen, the samples were refrigerated at −20°C. The fecal samples were dried at 65℃ for 72h, crushed, and screened through a 1mm sieve to be stored and further chemical analyses [35].

The method for analyzing CP (method No. 976.05), DM (method No. 930.15), EE (method No. 973.18), Ca, and P (method 935.13) in feed follows the approach outlined by the Association of Official Analytical Chemists ( [3]). The same method was used to analyze the chemical composition of goat manure. The method for analyzing NDF in feed follows the approach outlined by the method of Van Soest et al. [66] without thermostable α-amylase but uses sodium sulfite and NDF is expressed without residual ash. Following the methodology described by Van et al. [65] the endogenous indicator acid-insoluble ash (AIA) was employed to calculate apparent nutritional digestibility (Quantify digestibility).

### Rumen fermentation parameters

On the final day of the experiment, 2 h after morning feeding, the method of Long et al. [44], 6 goats were randomly selected and rumen fluid was collected from each goat through the mouth and esophagus using a vacuum pump catheter device. The pH value of the collected rumen fluid was analyzed immediately. In addition, the concentration of NH_3_-N of rumen fluid was analyzed. Notably, the remaining rumen fluid will be rapidly snap-frozen and stored in two portions, one at −20°C and the other at −80°C. After several days, gas chromatography (GC) was used to analyze the content of VFA in rumen fluid that had been kept at −20°C kept at −20°C underwent. The method of preparing rumen fluid samples for gas chromatography analysis was derived from the study by Han et al. [19].

The GC–MS analysis was performed on a trace 1300 gas chromatograph (Thermo Fisher Scientific, USA). Helium was employed as the carrier gas at 1 mL/min on a capillary column Agilent HP-INNOWAX (30 m × 0.25 mm ID × 0.25 μm) that was attached to the GC–MS. Next, with an injection volume of 1 μL and an injector temperature of 250 °C, the injection was made in split mode at a ratio of 10:1. The temperature of the contact and the ion source were 250 °C and 300 °C, respectively. Subsequently, the temperature increase in the column was programmed to start at 90 °C and climb to 120 °C at 10 °C/min, 150 °C at 5 °C/min, and 250 °C at 25 °C/min. The temperature was then maintained for 2 min (a total of 15 min of operation time). Thermo Fisher Scientific, USA's ISQ 7000 was used for the electron impact ionization technique of mass spectrometry to detect metabolites. An electron energy of 70 eV was employed in the single ion monitoring (SIM) mode. [22, 74].

### Serum parameters

On the day of the end of the experiment, blood was drawn from the jugular vein before feeding. From every goat, 10 ml of blood were drawn. Before analysis, serum was separated from blood and kept at −20°C. The levels of Immunoglobulin G (IgG), Immunoglobulin M (IgM), Immunoglobulin A (IgA), Interleukin-6 (IL-6), Interleukin-2 (IL-2), Interferon-gamma-γ (INF-γ), Tumor necrosis factor-α (TNF-α), Growth hormone (GH), Glutamic oxalacetic transaminase (ALT), andAlanine aminotransferase (AST) were analyzed using a microplate reader (ELX800, BioTek, USA) following the detailed procedures provided by the respective ELISA kits (Haoyuan Biotechnology Co., Ltd., Yibin, China).

### Rumen microbial profiles

Samples of rumen fluid were collected, snap-frozen, and kept at −80 °C. Bacterial DNA was recovered from the Rumen fluid (MagPure Soil DNA LQ Kit, D6356-02, Magen, Hilden, Germany). Following this step, using agarose gel electrophoresis and a NanoDrop 2000 spectrophotometer (Thermo Fisher Scientific, Waltham, MA, USA), the concentration and integrity of the DNA were determined. The PCR process amplified the V3-V4 hypervariable region of the bacterial 16S rRNA gene, employing primers (343F: 5′-TACGRAGCAGCAG-3′; 798R: 5′-AGGGTATCTAATCCT-3′) in a 25 μL reaction. The reverse primer, which included the sample barcode, was utilized, and each primer was connected to an Illumina sequencing adapter individually.

The Amplicon quality has been determined using gelatin electrophoresis. Subsequently, The Qubit dsDNA kit was utilized to quantify the outcomes after the PCR products were purified using Agencourt AMPure XP beads (Beckman Coulter Co., USA). The concentrations were adjusted thereafter to get ready for the sequencing procedure. The sequencing was conducted on an Illumina NovaSeq 6000 platform (Illumina Inc., San Diego, CA; OE Biotech Company, Shanghai, China), using two paired-end read cycles, each with a length of 250 bases.

The raw sequencing data was in FASTQ format. Subsequently, the obtained data underwent preprocessing using the Cutadapt software to detect and cut off the joints. Following this step, QIIME2 and DADA2 were employed to filter, denoise, merge, and detect and remove chimeric reads according to the methods described by Callahan et al. [9] and Bolyen et al. [7]. The representative readings and the ASV abundance table are finally output by the software. Using the QIIME2 package, the representative read of each ASV was chosen. Using the default parameters of q2-feature-classifier, all representative reads were annotated and blasted against Silva database Version 138 (or unity) (16s/18s/ITS rDNA). The calculation methods of *Chao1 index*, *Simpson index*, and *Shannon index* in α-diversity analysis were based on the methods of Hill et al. [21] and Chao et al. [11], respectively. Phylogenetic tree construction and unweighted Unifrac Principal coordinates analysis (PCoA) was conducted using the Unifrac distance matrix generated by QIIME software. OE Biotech Co., Ltd. handled the sequencing and analysis of the 16S rRNA gene amplicon (Shanghai, China).

### Statistical analysis

Initially, Excel 2021 was used to arrange and record all of the raw data that was gathered for this study, and the Shapiro–Wilk test was originally used to confirm that the data had a normal distribution. Subsequently, with the recorded data, statistical analysis was performed using SPSS 26.0 software. The data analysis includes one-way ANOVA and multi-covariate ANOVA with a general linear model (GLM module). Multiple comparisons and tests for significant differences were conducted using the LSD method and the Duncan's test. The means and standard error of the means (SEM) were used to express each result. In this study, *P* < 0.05 was regarded as statistically significant, and *P* < 0.01 was regarded as extremely significant.

## Results

### Organic acid, NH_3_-N, pH, and microbial population in FTMR

Table 2 shows that the content of NH_3_-N, yeasts, and molds in the CON group was significantly higher (*P* < 0.01) than those of the TCHMR group. On the other hand, the content of WSC, lactic acid, acetate, lactic acid/acetate, and LAB in the TCHMR group was significantly higher (*P* < 0.01) than those of the CON group.

**Table 2:** Organic acid, NH_3_-N, pH, and microbial population in FTMR (*n* = 6)

Item	Group		SEM	*P*-value
	CON	TCHMR		
pH	4.22	4.16	0.02	0.08
NH_3_-N (g/100g, DM)	7.96^**a**^	1.05^**b**^	1.04	< 0.01
WSC (mg/g)	25.78^**b**^	38.39^**a**^	1.92	< 0.01
Lactic acid (mg/g, DM)	8.08^**b**^	14.35^**a**^	0.96	< 0.01
Acetate (mg/g, DM)	1.75^**b**^	2.27^**a**^	0.08	< 0.01
Propionate (ug/g, DM)	3.18^**b**^	7.06^**a**^	0.59	< 0.01
Butyrate (ug/g, DM)	1.00	1.05	0.02	0.14
Lactic acid/Acetate ratio	4.63^**b**^	6.34^**a**^	0.27	< 0.01
LAB (log_10_, cfu/g, FM)	4.13^**b**^	10.72^**a**^	0.99	< 0.01
Yeasts (log_10_, cfu/g, FM)	8.57^**a**^	6.71^**b**^	0.28	< 0.01
Molds (log_10_, cfu/g, FM)	2.51^**a**^	2.29^**b**^	0.03	< 0.01

*CON* the formula of the control group remained unchanged but had been fermented, *TCHMR* Traditional Chinese herbal medicine residues group, *FM* Fresh matter, *DM* Dry matter, *cfu* colony-forming unit, Log_10_, cfu/g, *FM*, the total number of microbial communities

Different lowercase superscript letters (e.g., a, b) within the same line denote significant (*P* < 0.05) or highly significant (*P* < 0.01) differences. Please refer to the *p*-values provided in each table for detailed information. SEM, standard error of the mean. The remaining tables in this paper have the same data description methodology

### Growth performance and economic benefits

Effects of supplementing 40% TCHMR to the diet of Guizhou black male goats on growth performance (Table 3). Compared with the control group, TCHMR significantly increased DMI and feed weight gain cost (*P* < 0.01). Furthermore, the TWG, ADG and weight gain of the TCHMR group were also significantly increased (*P* < 0.05). Notably, the ADG of TCHMR increased by 25.53% and the weight gain benefit increased by 25.51% in comparison to the CON group (*P* < 0.05).

**Table 3:** Effects of 40% TCHMR in FTMR diets on growth performance and economic benefits of Guizhou black male goats

Item	Group		SEM	*P*-value
	CON	TCHMR		
DMI, (kg)	0.97^**a**^	1.07^**b**^	0.01	< 0.01
IBW, (kg)	22.10	21.95	0.82	0.99
FBW, (kg)	30.27	31.70	0.17	0.57
TWG, (kg/one goat)	8.17^**b**^	10.26^**a**^	0.46	0.02
ADG, (g/d)	136.18^**b**^	170.94^**a**^	7.74	0.02
FCR	7.14	6.66	0.25	0.35
Live goat real-time price, (CNY/kg) ^**a**^	40	40	-	-
Feed cost, (CNY/kg)	2.72	1.87	-	-
Feed weight gain cost, (CNY/kg)	21.89^**a**^	16.87^**b**^	0.82	< 0.01
Weight gain benefit, (CNY/one goat)	160.38^**b**^	201.29^**a**^	9.12	0.02

*FCR* Feed conversion ratio, *Kg/one goat* Kilograms per black goat, *CNY/kg* CNY per kg, *CNY/one goat* CNY per black goat, *IBW* Average initial body weight, *FBW* Average final body weight, *TWG* Average total weight gain

^a^ The current prices for live goats and feed raw materials (for example, TCHMR priced at 70 CNY per ton) reflect the prevailing market rates in Guizhou Province, China

ADG = (end of test weight − beginning of test weight)/number of feeding days; FCR = DMI / ADG; DMI (kg/d) = [feeding amount (kg) × diet DM (%) − leftover (kg) × leftover DM (%)] / [number of black goats per group × number of trial days (d)]

Feed weight gain cost (CNY/kg) = total feed intake × feed unit price (CNY) / total weight gain; Weight gain benefit (CNY/one goat) = (real-time price of live black goat − feed weight gain cost) × total weight gain

### Apparent digestibility

Compared with the CON group, it could be indicated from Table 4 that 40% TCHMR into the diets of Guizhou black male goats significantly improved (*P* < 0.01) the apparent digestibility of DM, CP, and NDF.

**Table 4:** Effects of 40% TCHMR in FTMR diets on apparent digestibility of Guizhou black male goats (%)

Item	Group		SEM	*P*-value
	CON	TCHMR		
DM	72.49^**b**^	83.87^**a**^	1.77	< 0.01
EE	70.22	70.61	0.69	0.80
CP	75.30^**b**^	83.05^**a**^	1.31	< 0.01
NDF	65.85^**b**^	73.46^**a**^	1.50	< 0.01

### Ruminal fermentation parameters

Effects of 30% TCHMR on ruminal fermentation parameters of Guizhou black male goats are shown in Table 5. The incorporation of 30% TCHMR in Guizhou black male goat diets could significantly improve (*P* < 0.01) the value of pH. Incredibly, findings from TCHMR showed that it lowers (*P* < 0.01) the content of acetate in the rumen when compared to the CON group. In addition, the content of isobutyrate and isovalerate in the TCHMR group were also significantly lower (*P* < 0.05) than those in the CON group.

**Table 5:** Effects of 40% TCHMR in FTMR diets on ruminal fermentation indexes of Guizhou black male goats

Item	Group		SEM	*P*-value
	CON	TCHMR		
pH	6.34^**b**^	6.67^**a**^	0.08	0.03
NH_3_-N, (mg/dL)	12.31	11.16	0.65	0.39
TVFA, (mmol/L)	54.53	51.31	3.64	0.67
VFA, (% of the total TVFA)				
Acetate	70.13^**a**^	67.93^**b**^	0.92	< 0.01
Propionate	13.92	17.43	1.01	0.08
Butyrate	11.87	14.20	0.67	0.08
Isobutyrate	1.65^**a**^	1.12^**b**^	0.13	0.03
Isovalerate	1.21^**a**^	0.88^**b**^	0.08	0.04
Valerate	1.01	0.98	0.03	0.73
A/P	5.20	4.22	0.28	0.07

*TVFA* Total volatile fatty acids, *A/P* Acetate/propionate

### Serum parameters

**Fig. 1:**
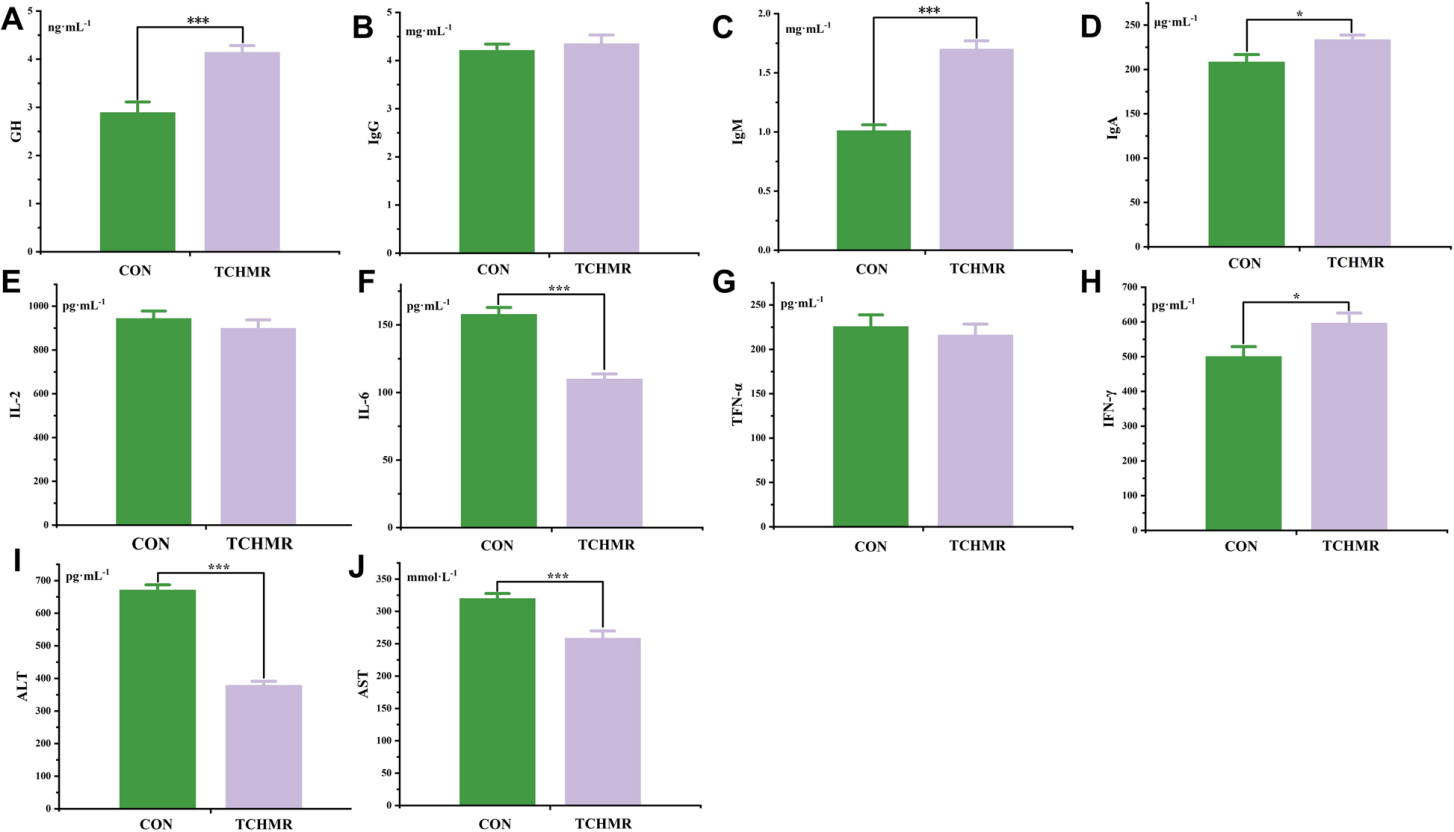
Effects of 40% TCHMR in FTMR diets on hematology indexes of Guizhou black male goats. **A** level of GH; **B**, level of IgG; **C**, level of IgM; **D**, level of IgM; **E**, level of IL-2; **F**, level of IL-6; **G**, level of TNF-α; **H**, level of INF-γ; level of ALT; **J**, level of AST. *P < 0.05; **P < 0.01; ***P < 0.001

Effects of 30% TCHMR on hematology index of Guizhou black male goats are shown in Fig. 1. The levels of GH and IgM in the TCHMR group were significantly higher (*P* < 0.001) than those in the CON group. Meanwhile, the levels of IgA and IFN-γ in the TCHMR group were also significantly higher (*P* < 0.05) than those in the CON group. Notably, TCHMR significantly reduced (*P* < 0.001) the levels of ALT and AST in Guizhou black male goats.

### Rumen microbial profiles

**Fig. 2:**
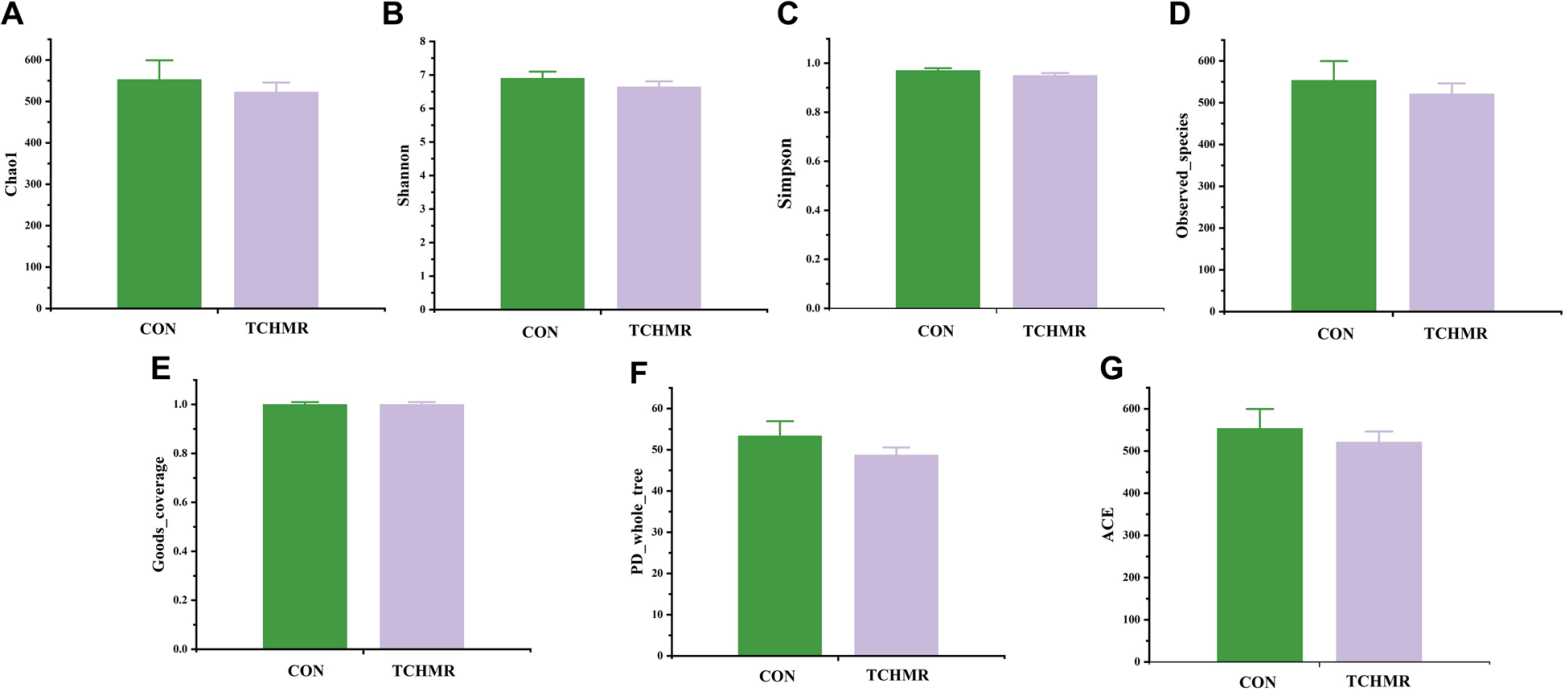
Effects of 40% TCHMR in FTMR diets on the alpha diversity index of ruminal microbiota in Guizhou black male goats. **A** *Chao1* index; **B**, *Shannon* index; **C**, *Simpson*index; **D**, *Observed_species* index; **E**, *goods_coverage* index; **F**, *PD_whole_tree* index; **G**, *ACE* index. *P < 0.05; **P < 0.01; ***P < 0.001

16S rDNA sequencing was used to determine the ruminal microbiota. The incorporation of TCHMR in goat diets couldn’t statistically significantly impact the indices of *Chao1*, *Shannon*, *Simpson*, *ACE*, *goods_coverage*, *observed_species*,and *PD_whole_tree* in the alpha diversity analysis (Fig. 2). The results of the data demonstrated that the microbial coverage was broad, the distribution was even, the sequencing depth was appropriate, the data dependability was strong, and the information gleaned from the samples was sufficient to characterize the microbial composition.

**Fig. 3:**
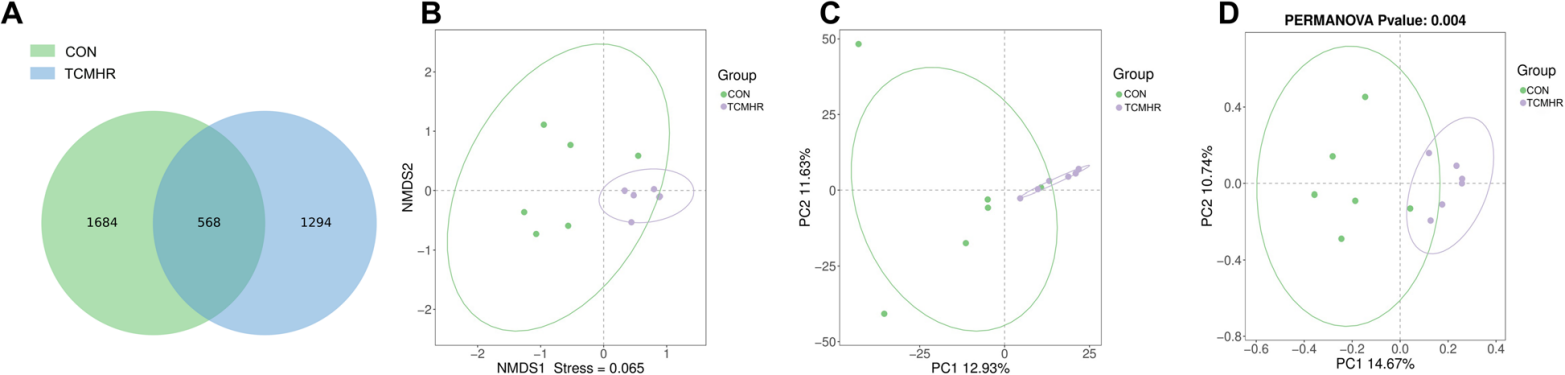
Effects of 40% TCHMR in FTMR diets on ASV and beta diversity in Guizhou black male goats. **A** ASV-Venn; **B**, non-metric multidimensional scale analysis (NMDS); **C**, Principal component analysis (PCA); **D**, Principal coordinate analysis (PCoA)

The ASV-Venn results showed that ASV-specific bacteria in the CON group accounted for 47.49% (1684) of the total ASV, and ASV-specific bacteria in the TCHMR group accounted for 36.49% (1294) of the total ASV. Furthermore, ASV bacteria shared by both groups accounted for 16.02% (568) of the total ASV (Fig. 3A). NMDS analysis showed that stress = 0.065 < 0.2 between two groups, demonstrating the stability of the model and the accuracy of the data (Fig. 3B). Notably, it could be shown from the graphs produced by the NMDS, PCA (Fig. 3C), and PCoA (Fig. 3D) analyses that both groups had large contribution rates and the sample distances between them were considerably scattered. This revealed that TCHMR changed the composition of goat ruminal microbiota.

**Fig. 4:**
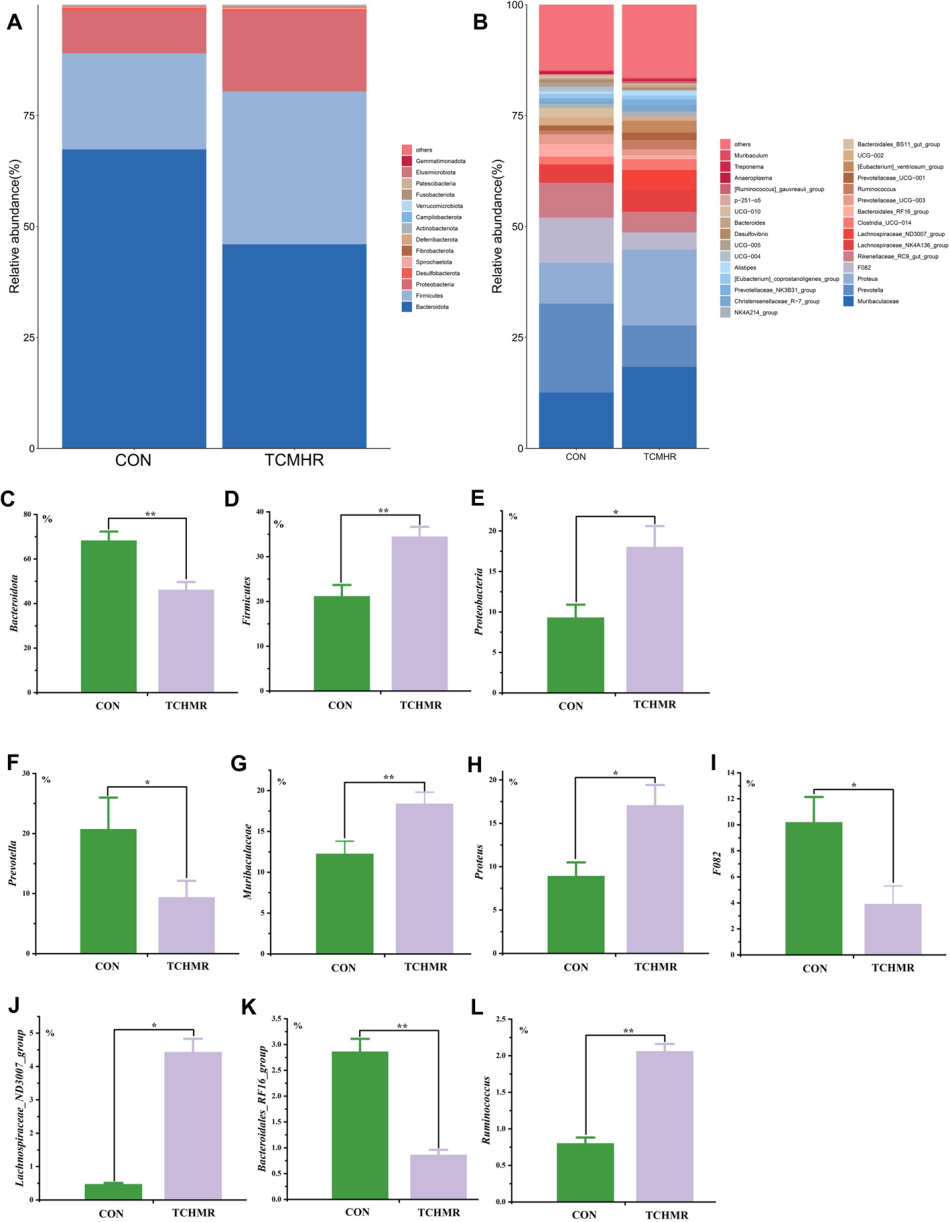
Effects of TCHMR on the relative abundance of microbiota at the phylum and genus levels in Guizhou black male goats. **A** microbiota taxonomic profiling of rumen microbiota at the phylum level; **B**, microbiota taxonomic profiling of rumen microbiota at the genus level; **C**, relative abundance of *Bacteroidota*; **D***,* relative abundance of *Firmicutes;* **E***,* relative abundance of *Proteobacteria*; **F**, relative abundance of *Prevotella*; **G**, relative abundance of *Muribaculaceae*; **H**, relative abundance of *Proteus*; **I**, relative abundance of *F082*; **J**, relative abundance of*Lachnospiraceae_ND3007_group*; **K**, relative abundance of *Bacteroidales_RF16_group*; **L**, relative abundance of *Ruminococcus*. *P < 0.05; **P < 0.01; ***P < 0.001

The abundance of bacteria below 0.1% and unclassified was not shown in the taxonomic composition diagram of the ruminal microbiota at the phylum (Fig. 4A) and genus (Fig. 4B) levels. More than 98% of the ruminal microbiota were composed of the three primary dominat bacterial phyla: *Firmicutes*, *Proteobacteria*, and *Bacteroidetes*. Interestingly, TCHMR significantly increased the abundance of *Firmicutes* (*P* < 0.01) and *Proteobacteria* (*P* < 0.05), whereas on the contrary, it significantly decreased (*P* < 0.01) the abundance of *Bacteroidota* (Fig. 4). At the genus level (Fig. 4), *Prevotella*, *Muribaculaceae*, *Proteus*, *F082*, and *Rikenellaceae_RC9_gut_group* were the main dominant genera, accounting for more than 53% of the total ruminal microbiota. TCHMR significantly reduced (*P* < 0.05) the abundance of *Prevotella, Proteus, Lachnospira ceae_ND3007_group,* and*F082*. Meanwhile, TCHMR significantly increased (*P* < 0.01) the abundance of *Muribaculaceae*, *Bacteroidales_RF16_group,* and *Ruminococcus*.

**Fig. 5:**
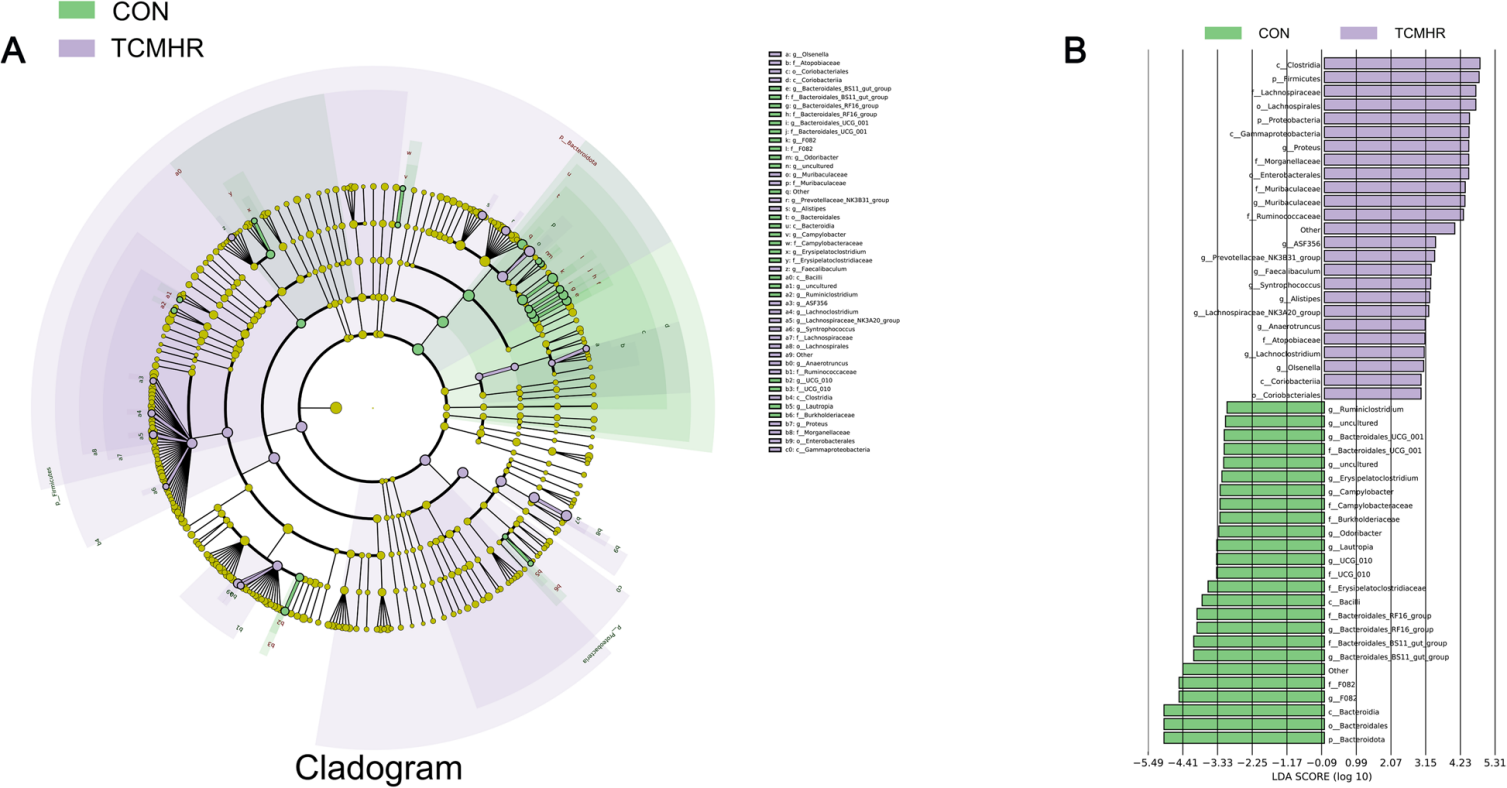
Linear discriminant analysis (LDA/ LEfSe) coupled with effect size measurements analyzes the differences between the two groups of microorganisms. **A** a histogram was generated based on the distribution of LDA values (LDA score > 2), and the length of the bar shows the abundance of various species; **B**, example diagram of annotated branches of different species, different colors represent different groups. The light purple nodes and light green nodes represent significantly different species with relatively high abundance in the light purple group and light green group respectively. The yellow nodes represent species that have no significant difference in the comparison between the two groups. The node diameter varied proportionally with the relative abundance. The nodes within each layer represent phylum/class/order/family/genus, progressing from the innermost to the outermost layers. The species denoted by English letters in the figure are elucidated in the legend positioned on the right

The LEfSe analysis properly identified the significant bacterial groups associated with the two groups using all available microbial data. The main differential species between the two groups could be clearly drawn from the Cladogram and LDA graphs (Fig. 5). Each of the TCHMR and CON groups obtained 25 distinct, high-abundance bacterial colonies and they were all different, indicating that TCHMR reorganized the goat ruminal microbiota composition.

**Fig. 6:**
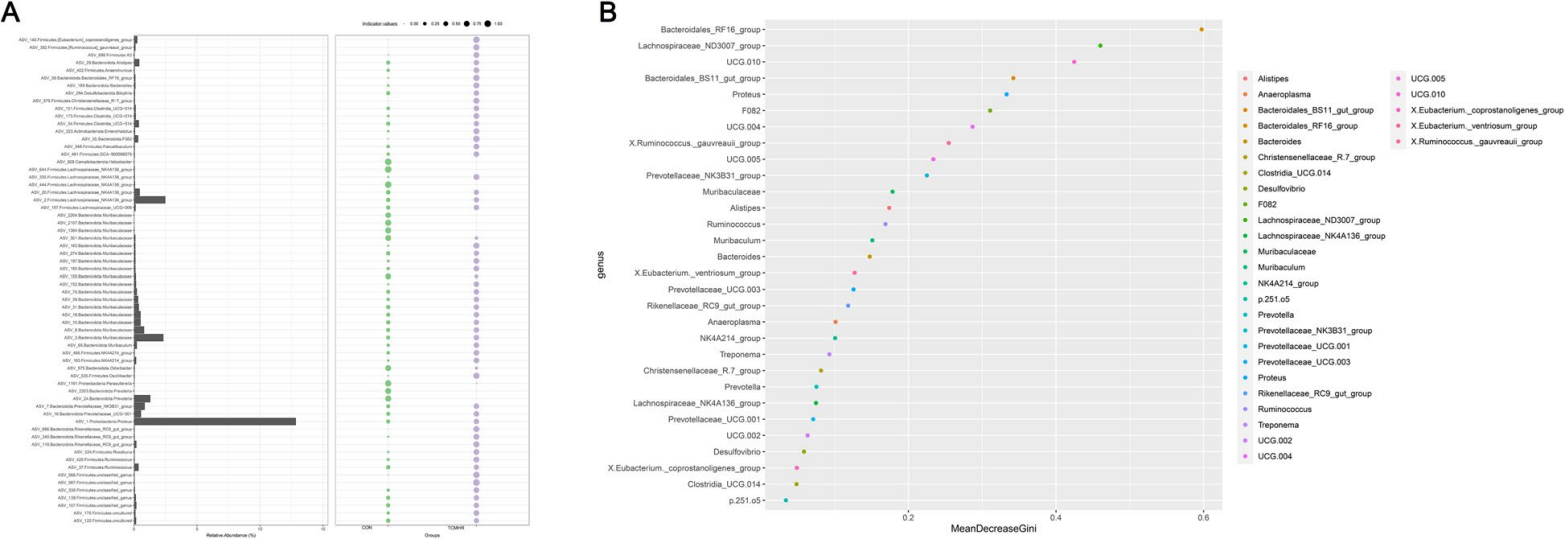
Indicator analysis and random forest analysis analyzed the differences between the two groups of microorganisms.** A** the first, second, and third columns shown in the figure were species phylum level annotation information, and species genus level annotation information. The histogram illustrates the abundance of each ASV, while the abscissa and the size of the bubbles in the figure denote the sample groups and the indicator values of each species within the sample group, respectively. (The size of the bubbles indicates the relative abundance of species within the group, with significance criteria set at < 0.05); **B**, the abscissa represents the measure of importance, and the ordinate displays the species names arranged according to their respective importance levels

The indicator species figure (Fig. 6) illustrates biological genera, species, or communities that potentially exert a significant influence on the growth environment within a specific area. Through Indicator analysis (Fig. 6A), it could be concluded that *ASV_2.Firmicutes.Lachnospiraceae_NK4A136_group*, *ASV_3.Bacteroidota.Muribaculaceae*, and *ASV_1.Proteobacteria.Proteus* had the highest abundance and the greatest impact on the growth environment. From the changes in the indicator values of the two groups of species in the bubble chart, it could be found that TCHMR altered the composition of ruminal microbiota in comparison to the CON group, which led to distinct changes in the effects of various microbial species on the growth environment. From the random forest analysis (Fig. 6B), the importance ranking of ruminal microbiota genera was obtained. *Bacteroidales_RF16_group* was the most important genera, followed by *Lachnospiraceae_ND3007_group*, *UCG.010*, and so on.

### Correlation analysis

**Fig. 7:**
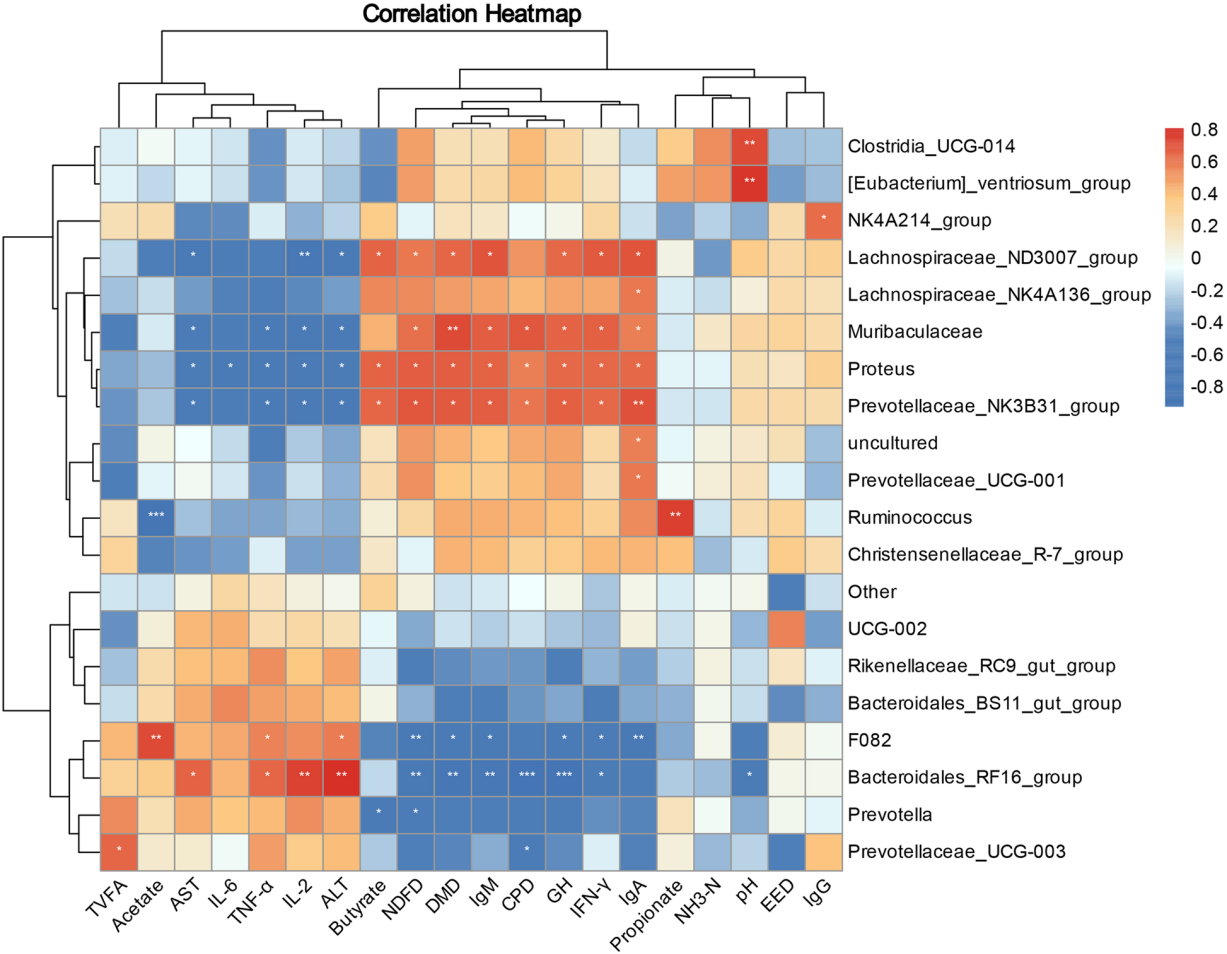
Rumen fermentation parameters, serum biochemical indicators, apparent digestibility of nutrients and correlation analysis with microorganisms. A TVFA, total volatile fatty acids; DMD, apparent digestibility of dry matter; CPD, apparent digestibility of protein; EED, apparent digestibility of crude fat; NDFD, apparent digestibility of NDF. Red denotes a positive correlation, while blue indicates a negative. *P < 0.05, **P < 0.01, ***P < 0.001

The Pearson correlation calculation method was employed to reveal the correlation between rumen microbiome composition and serum biochemical indicators, rumen fermentation, and apparent digestibility of nutrients (Fig. 7). pH changes were negatively (*P* < 0.05) correlated with *Bacteroidales_RF16_group* and positively (*P* < 0.01) correlated with *Clostridia_UCG-014* and *[Eubacteria]_ventriosum_group*. *Lachnospiraceae_ND3007_group*, *Proteus*, and *Prevotellaceae_NK3B31_group* were positively (*P* < 0.05) correlated with butyrate, NDF, DMD, IgM, GH, and IFN-γ, and negatively (*P* < 0.05) correlated with ALT and AST. *Muribaculaceae* was positively (*P* < 0.01) correlated with DMD, while *Prevotellaceae_NK3B31_group* was positively (*P* < 0.01) correlated with IgA. In addition, *Lachnospiraceae_ND3007_group* was negatively (*P* < 0.01) correlated with IL-2. *Ruminococcus* had a positive (*P* < 0.01) correlation with propionate. In contrast, *F082* was negatively (*P* < 0.01) correlated with IgA and NDFD, and positively (*P* < 0.01) correlated with acetate. *Bacteroidales_RF16_group* was negatively (*P* < 0.01) correlated with IgM, DMD, and NDFD, while, it was positively (*P* < 0.01) correlated with ALT and IL-2. Notably, *Bacteroidales_RF16_group* was extremely significantly positively (*P* < 0.001) correlated with ALT, and extremely significantly negatively (*P* < 0.001) correlated with GH and CPD. Interestingly, *Ruminococcus* had an extremely significant negative (*P* < 0.001) correlation with acetate.

### 16S-based KEGG function prediction

**Fig. 8:**
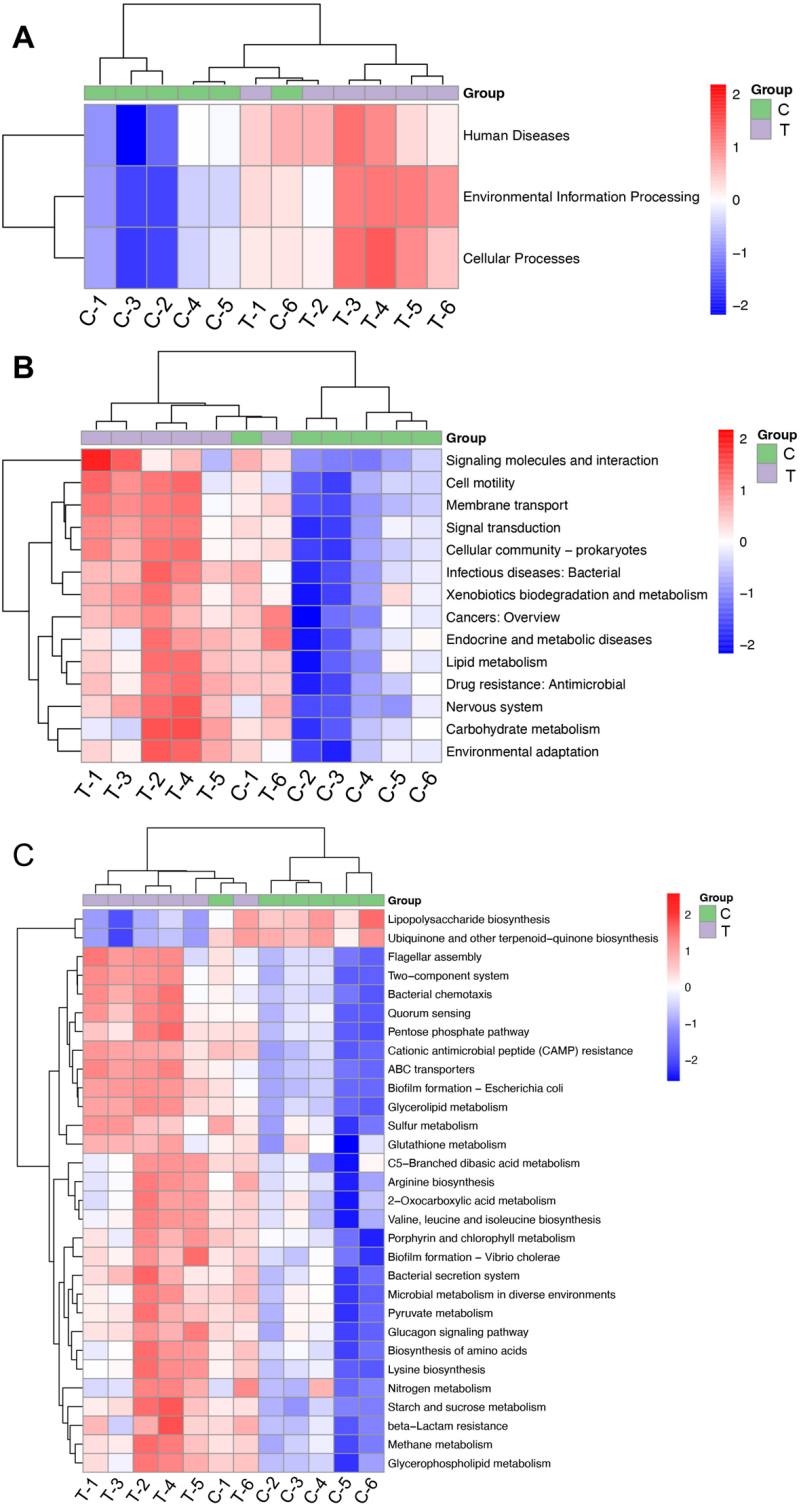
KEGG differential result heatmap obtained by Wilcoxon algorithm. **A** KEGG heat map of the first-level (level 1) function prediction; **B**, KEGG heatmap of the second-level (level 2) function prediction; **C**, KEGG heatmap of the third-level (level 3) function prediction

The three-level functional prediction results of the Kyoto Encyclopedia of Genes and Genomes (KEGG) based on the Wilcoxon algorithm are shown in Fig. 8. Compared with the CON group, at the first level of KEGG (Fig. 8A), we discovered that TCHMR encouraged the enrichment of microbial flora in pathways of cellular processes, environmental information processing, and human diseases. In subsequent studies, we identified the top 14 information pathways in the second level of KEGG (Fig. 8B), including lipid metabolism, nervous system, and carbohydrate metabolism, and so on. Unexpectedly, we found that TCHMR upregulated almost all 14 pathways analyzed in the figure. Compared with the CON group, the microbial enrichment of the TCHMR group was more advantageous in these 14 pathways. Moreover, in functional prediction, we continued to conduct an in-depth analysis of the top 30 information pathways in the third level of KEGG (Fig. 8C), including signaling pathways such as lipopolysaccharide biosynthesis, glycerolipid metabolism, and biosynthesis of amino acids. Interestingly, TCHMR downregulated the signaling pathways of Lipopolysaccharide biosynthesis and Ubiquinone and other terpenoid − quinone biosynthesis. Additionally, TCHMR upregulated all remaining signaling pathways.

## Discussion

### Effect of 40% TCHMR in FTMR diets on feed quality

The pH value of well-fermented feed is generally 4.2 or lower [33]. The number of LAB attached to the feed surface produces a large number of organic acids (mainly LA) by converting carbohydrates, which is crucial to the initial fermentation of the feed. Sufficient WSC can provide sufficient carbon sources for LAB, accelerate the production of LA, accelerate the acidification of feed, and reduce the pH value to inhibit microbial activity [36, 70]. In our study, the levle of WSC, LAB, and LA in theTCHMR group were higher than those in the CON group.This illustrates that incorporating TCHMR into the feed supplied sufficient carbon sources for lactic acid bacteria, thereby increasing the production of LA, lowering the pH to below 4.2, and ultimately yielding superior fermentation effects.

On the other hand, acetate is another key indicator for evaluating the quality of fermented feed. Its high content is conducive to the growth of undesirable microorganism *Clostridia*, which may cause large amounts of protein to be degraded [67]. However, Protein degradation transforms true protein (TP) into non-protein nitrogen (NPN) such as free amino acid-N (FAA-N) and NH_3_-N [68]. The NH_3_-N content is the main indicator to reveal the decomposition efficiency of protein to peptides in fermented feed [27]. Typically, high-quality fermented feeds contain higher LA and lower levels of H_3_-N and acetate, resulting in a higher lactic acid/acetate ratio [33]. We discovered that feeds incorporating TCHMR exhibited a greater concentration of acetate and a diminished concentration of NH_3_-N. Nonetheless, the ultimate ratio of LA to acetate was significantly elevated compared to that in the CON group. Based on this result, we theorized that this could be due to the higher content of WSC in the TCHMR group. This occurs as lactic acid bacteria in the feed utilize WSC to facilitate the substantial production of organic acids (including LA, acetate, propionate, and so on). Moreover, the higher content of an antifungal agent (propionate) was observed in the TCHMR group providing further elucidation for this inference. Interestingly, the content of yeast in the CON group was significantly higher than those in the TCHMR group.

Furthermore, yeast metabolizes LA and WSC into CO_2_ and water under aerobic conditions, which is the primary cause of aerobic deterioration in fermented feed [20]. Consequently, we believe that yeast grows more competitively than lactic acid bacteria in the CON group environment and depletes LA and WSC from the feed in significant quantities, creating more favorable conditions for the growth of undesirable microorganisms. However, it is important to note that the elevated levels of molds observed in the CON group in my study provide additional evidence supporting our speculation.

### Effect of 40% TCHMR in FTMR diets on growth performance, economic benefits, and apparent digestibility

In recent years, the investigation into the utilization of Chinese herbal medicine residues as feed additives or substitutes for antibiotics has emerged as a prominent focus of current research [1]. Previous research has shown that the incorporation of 15% *Ruellia tuberosa* medicinal residue in white cashmere goat diets could significantly increase the DMI and ADG of the goats [50]. In our study, we observed that incorporating 40% TCHMR into goat feed led to heightened DMI, TWG, and ADG, while simultaneously reducing FCR. Our findings closely resemble those reported in recent studies exploring the effects of traditional Chinese medicine residues on beef cattle [41]. This may be due to the medicinal properties of TCHMR itself and its high nutrient content.

From an economic perspective, the TCHMR used in this study is highly affordable, priced at only around 70 CNY per ton. The cost per kilogram of FTMR feed supplemented with TCHMR was 31.25% lower than that of the FTMR in the CON group. Additionally, the feed weight gain cost in the TCHMR group was 22.93% lower than in the CON group. Finally, the weight gain benefit in the TCHMR group was 25.51% higher than that in the CON group. Consequently, based on the analysis of goat growth performance, feed costs, and overall economic benefits, TCHMR emerges as a promising and cost-effective new feed resource with great development potential.

The apparent digestibility of nutrients serves as a direct indicator of diet utilization and digestibility. It was concluded by Meng et al. [50] that supplementing feed with *Ruellia tuberosa* enhanced the apparent digestibility of DM, NDF, and CP in white cashmere goats. We obtained similar results in our study, where the inclusion of TCHMR increased the apparent digestibility of DM, NDF, and CP in goats. Nevertheless, it is noteworthy that the results of our study diverge from those reported by Liu et al. [41].We posit that this phenomenon may be attributed to the fermented TCHMR, which enhances feed quality, reorganizes ruminal microbiota in goats, increases ruminal microbiota activity, and augments the activities of proteases and cellulase, thereby ultimately promoting the digestion and absorption of nutrients. Indeed, the enhanced apparent digestibility observed in goats also offers an additional explanation for the research findings suggesting that TCHMR improves the growth performance of goats.

### Effect of 40% TCHMR in FTMR diets on ruminal fermentation indexes

Ruminal pH serves as a crucial metric for assessing the capacity of TCHMR to instigate alterations in the rumen environment through their impact on ruminal microbes and fermentations [32]. It was concluded by Jasmin et al. [26] demonstrated that the rumen pH value should be between 5.5 and 7.0. Anything exceeding 7.0 or falling below 5.5 is deemed abnormal.The optimal pH range for the growth of ruminal microbiota is 6.5 ~ 7.0 [10]. In our study, although the ruminal pH value of the TCHMR group was significantly higher than that of the CON group. However, the pH values remained within the normal range for the rumen environment and the growth of ruminal microbiota was also within the optimal pH value range. Conversely, the value of pH in the CON group was below 6.5, which may affect the growth of certain ruminal microbiota. This result indicates that under the current feeding conditions, the incorporation of TCHMR in goat diets could sustain normal ruminal fermentation without inducing rumen acidosis.

On the other hand, in the results of this study, we also found that the content of acetate TCHMR was significantly lower than that of the CON group. This phenomenon could be attributed to the interconversion of acetate and butyrate within the rumen, approximately 28% of the acetate present will not be absorbed in its original form [31]. At this time, acetate is used by microorganisms to produce butyrate through the pathway of acetyl- CoA transferase or butyryl-CoA transferase (Such as butyryl-coA + acetate → butyrate + acetyl- coA), instead of producing acetoacetyl-CoA from two acetyl-CoA [15, 18]. Then, ruminal microbiota can promote microbial metabolism through this energy consumption process, and butyrate is continuously converted from acetate [18]. Additionally, TCHMR has the potential to alter the rumen fermentation pattern of goats, transitioning it from acetate to propionate [77]. In VFA, when acetate reaches the peripheral circulation, propionate will be largely converted to glucose by the liver, serving as a primary energy source for the body [4]. In this study, we discovered that the level of acetate in the TCHMR group were decreased, while propionate and butyrate tended to increase. This result will provide a more reasonable explanation for our speculation. Furthermore, the value of A/P was decreased in the TCHMR group showing that TCHMR improves the feed energy utilization efficiency [40]. This can explain the higher growth performance of the TCHMR group under the conditions of this experiment because butyrate and propionate are more conducive to improving the growth performance of goats.

### Effect of 40% TCHMR in FTMR diets on Serum biochemical indexes

One of the most crucial indicators in animal research is the serum biochemical indicator, which reflects the nutritional status, metabolic state, and overall health of the animal [57]. In this study, the level of GH in the TCHMR group were significantly higher than those in the CON group. This result indicates that TCHMR produces better results in fostering goat growth and development as compared to the CON group. The immunoglobulins produced in the first immune response serve as the main antibodies in the body (including IgA, IgG, and IgM). These antibodies also play a pivotal role in the secondary immune response against pathogens [12]. Type II interferon (IFN-γ) is produced by NK cells and T lymphocytes and plays an important role in all stages of the immune response [59]. IFN-γ is very important in the anti-viral process. It has the function of inhibiting viral proliferation, activating T cells to produce cytokines, and improving the killing activity of cytotoxic T lymphocytes [34].

In our study, it was found that TCHMR significantly increased the levels of IgM, IgA, and IFN-γ in goats. Moreover, we also observed that TCHMR significantly reduced the levels of IL-6 in goats. This may be attributed to the composition of the TCHMR we added, primarily comprising *Panax ginseng*, *Codonopsis pilosula*, *Astragalus membranaceus*,*Eleutherococcus senticosus*, and so on. Because*Panax ginseng* [55], *Codonopsis pilosula* [42], *Astragalus membranaceus* [76], and *Eleutherococcus senticosus* [37] have all been demonstrated in numerous prior studies to have immune-boosting, antioxidant, and anti-inflammatory qualities in animals. Nevertheless, the residue remaining after processing and extracting these Chinese herbal medicines into products still retains numerous medicinal effects. Feeding animals for a long time can achieve certain effects. Additionally, we speculated that the reduction in IL-6 levels observed in the TCHMR group may be attributed to the content of neutral polysaccharides in TCHMR. Because the neutral polysaccharides in ginseng can reduce the expression levels of IL-6, TFN-α, and interleukin (IL)−1β [71]. It is also significant that the activities of AST and ALT in serum are quite low under normal conditions. However, when liver tissue cells are damaged or lesions occur, and finally liver function is impaired [5]. Thus, AST and ALT are released into the bloodstream, causing an increase in the level of ALT and AST in the serum. Our study found that the level of ALT and AST in the TCHMR group were significantly lower than those in the CON group, so we believe that the incorporation of TCHMR in goat diets will not cause liver damage in goats under our experimental conditions.

### Effect of 40% TCHMR in FTMR diets on ruminal microbiota

In our study, at the phylum level, the main dominant ruminal microbiota in both groups were *Firmicutes*, *Bacteroidota*,and *Proteobacteria*, collectively representing over 90% of the total microbial population. Our data support the results reported by previous researchers Xue et al. [72] and Zhong et al. [76]. It is known that the function of *Firmicutes* is mainly to degrade fiber and cellulose [13]. However, the higher total flavonoid content of *Codonopsis pilosula* in TCHMR could increase the abundance of *Firmicutes* [28]. Hence, the higher apparent digestibility of NDF by TCHMR in this study will be better explained. On the other hand, the phylum *Bacteroidetes*assumes a crucial role in the degradation of proteins and carbohydrates, which has important implications for the digestive utilization of proteins and non-fibrous polysaccharides [13, 14]. Nevertheless, this is contradictory to the results in our study that the abundance of *Bacteroidetes* in TCHMR was lower than those in the CON group, but the apparent digestibility of CP was significantly higher than those in the CON group. We speculate that this effect may be attributed to TCHMR accelerates the rate of rumen absorption of CP in the feed. Therefore, the location of degradation and transformation of CP is shifted from the rumen to the intestine. However, the exact causes need to be further researched and explored. Moreover, it is also significant that the abundance analysis of pathogenic bacteria in the rumen. Elevated levels of the increased abundance of Proteobacteria (one phylum containing bacterial populations associated with pathogenicity) in the rumen may indicate an imbalance in the rumen microbiota, potentially leading to or exacerbating health issues such as rumen dysfunction, acidosis, inflammatory risks, metabolic disorders, and reduced fermentation efficiency ( [2, 61]). In this study, the abundance of *Proteobacteria* in the TCHMR group was significantly higher than those in the CON group. This may be due to the higher content of flavonoids in TCHMR [28]. However, based on our research findings, although the normal growth and rumen fermentation of the goats were unaffected under the conditions of this experiment, the increased abundance of *Proteobacteria* in the rumen suggests that TCHMR may have a negative impact on rumen health. For instance, will long-term feeding of TCHMR to goats result in a higher abundance of Proteobacteria in the rumen? Will the negative effects on the rumen become more pronounced? Ultimately, could the increase in the abundance of *Proteobacteria* eventually affect the growth, immunity, meat quality, and economic benefits of goats? Therefore, preventing these issues and ensuring the safety of TCHMR feed in long-term feeding conditions is an urgent challenge that we aim to address in the next phase of our research.

In our study, it was concluded that *Prevotella*, *Proteus*, *F082*, and *Muribaculaceae* were the dominant bacterial groups at the genus level. It is known that *Prevotella* is recognized to be primarily engaged in the degradation of proteins and starches. It also possesses enzymes that break down xylan and other hemicelluloses into short-chain fatty acids (SCFA) [16, 24]. In our study, TCHMR significantly reduced the abundance of *Prevotella*, but the apparent digestibility of CP was indeed improved, Thus, we can speculate that TCHMR altered the composition of ruminal microbiota and may have changed the location of CP degradation. To our knowledge, the abundance of *Prevotella* is significantly positively correlated with total VFA and acetate. However, it is possible that lower levels of isobutyrate and isovalerate are produced [70]. Our data support the findings of the above study, in which TCHMR reduced the abundance of *Prevotella*, ultimately resulting in lower levels of total VFA, acetate, isobutyrate, and isovalerate. In addition, we also found that *Prevotella* was negatively correlated with the levels of butyrate and the apparent digestibility of NDF (Fig. 5).

*Muribaculaceae* were called MIB (mouse intestinal bacteria) (GenBank accession number. AJ400263.1) or *Homeothermaceae* in previous reports [54], classified so far as S24-7 in SILVA [58] or *Porphyromonadaceae* in RDP [69]. *Muribaculaceae* is a recently described novel species that is widely distributed in the mouse intestine. A previous study by Ormerod et al. [54] indicated that *Muribaculaceae* contain numerous multifunctional carbohydrate-active enzymes, which they described as specialists in phytoglycans, α-glucans, and host glycans, respectively. Because *Muribaculaceae* can break down complex carbohydrates, such as polysaccharides and fiber, into SCFA like acetate, butyrate, and propionate. These metabolites provide energy to the host and promote intestinal health. In our study, the abundance of *Muribaculaceae* in TCHMR was significantly higher than those in the CON group, which may explain part of the reason for the higher content of propionate and the higher digestibility of NDF. However, currently, the majority of studies on *Muribaculaceae* are centered around mice, with limited research conducted on other mammals. Therefore, based on the findings presented in this study, it is necessary to further explore the mechanism and function of *Muribaculaceae* on goats.

In our results, TCHMR significantly increased the abundance of Proteus (Gram-negative bacteria), which belongs to *Proteobacteria*, but under the conditions studied in this article, no adverse effects on the growth and digestion of goats were observed. It was reported that F082 exhibits a positive correlation with propionate production [48, 49], but the findings of our study contradict this observation. The specific reasons require further research. *Ruminococcus* and *Lachnospiraceae*are key fiber-degrading bacteria in the rumen, supplying energy to the host through the degradation of plant cellulose and hemicellulose, and the subsequent production of SCFA [56, 64]. Moreover, they also possess diverse beneficial physiological functions, including enhancing immune regulation and reinforcing the intestinal barrier [6, 51]. In this study, TCHMR significantly increased the abundance of *Lachnospiraceae* and *Ruminococcus*. This potentially contributed to the higher apparent digestibility of NDF, the increased propionate and butyrate content, as well as the higher levels of related immune indicators.

## Conclusion

Under the conditions of this study, we tried to explore the effects of the TCHMR diet on the fattening effect and rumen health of Guizhou black male goats. Our research found that 40% TCHMR FTMR improved the quality of feed and the apparent digestibility of nutrients. Furthermore, TCHMR reduced FCR, increased ADG, and enhanced immunity in goats. Finally, TCHMR also reorganized ruminal microbiota composition and improved eventual economic benefits. In conclusion, the incorporation of TCHMR in goat diets is a low-cost and potentially unconventional feed development idea. So far, we have not found any negative effects of TCHMR on goat growth. Notably, TCHMR reorganized ruminal microbiota composition and appeared to increase the abundance of some harmful bacteria. In the current study, we believe that long-term feeding of TCHMR may negatively impact rumen function by promoting the growth of certain harmful bacteria, potentially compromising the healthy growth of goats. Consequently, in the future, we will further investigate the mechanism by which TCHMR affects the growth of harmful bacteria in the rumen to ensure the feed’s cost-effectiveness and safety. Additionally, we will conduct large-scale, representative production trials under different environmental conditions and develop TCHMR FTMR pellet feed to lay a solid foundation for promoting this product.

## Data Availability

The sequencing data of this study are deposited in the NCBI repository, accession number PRJNA1102846. Other data used to support the results of this study can be provided at the request of the corresponding author.
